# A Metasticker Composed of Indium-Tin-Oxide-Square-Fractal Rings for Broadband Absorption

**DOI:** 10.3390/ma19020297

**Published:** 2026-01-12

**Authors:** Min-Sik Kim, Won-Woo Choi, Yongjune Kim

**Affiliations:** 1 Department of Electrical Engineering, The University of Suwon, Hwaseong 18323, Republic of Korea; 2 Center for Advanced Meta-Materials, Daejeon 34103, Republic of Korea

**Keywords:** metasticker, metasurface absorber, square-fractal ring, genetic algorithm, broadband

## Abstract

This study proposes design and fabrication methods for an electromagnetic metasurface absorber (MA) that absorbs electromagnetic waves using a metasticker attached on a dielectric substrate blocked by a copper sheet. To guarantee a high design freedom as well as make the absorption bandwidth (BW) as broad as possible, a square-fractal ring is chosen as the metapattern, and its design is optimized using a genetic algorithm. To fabricate the square-fractal rings in a simple manner, an indium-tin-oxide film is cut by using a laser-cutting machine. Then, the metasticker is fabricated by assembling the metapatterns on a double-sided adhesive film which could be attached on the dielectric substrate using the opposite side of the film. From measured results of the finalized MA of which damaged regions caused by the laser-cutting process are compensated in the design process, a broad −10 dB reflectance BW is confirmed from 4.39 to 7.51 GHz of which the fractional BW is 52.44% for the normal incidence. Moreover, a fractional BW of 4.35% is measured in a wide incident angle range from 0° to 60° for both the transverse electric and the transverse magnetic polarizations simultaneously.

## 1. Introduction

Metasurface absorbers (MAs) are electromagnetic (EM) wave absorbers that utilize conductive metapatterns and dielectric materials that convert the incident EM energy into heat. For the metapatterns that utilize metals such as copper or gold, the absorptions occur dominantly by the dielectric losses induced by the electric (E) fields coupled with resonating electric currents. Based on the resonances, MAs can enhance the absorption levels, which can reach 100% at single [1,2,3] or dual frequencies [4,5,6] with narrow bandwidths (BWs). On the other hand, metapatterns composed of lossy materials such as carbon, graphene, silver nanoparticles, or indium tin oxide (ITO) absorb the EM waves via the ohmic losses induced by the resonant electric currents. Based on the low quality (Q) of the resonances, their fractional BWs can be extended over 100%, which is defined by 90% absorption [7,8,9,10,11]. Moreover, MAs can cover wide incident angle ranges of up to 60° for both the transverse electric (TE) and the transverse magnetic (TM) polarizations [12,13,14].

However, one of the drawbacks of MAs is that the fabrication process might be complex. For instance, a burdensome soldering of chip resistors is needed for MAs based on printed circuit boards for broadband [15,16] or wide-angle [14] absorptions. Even though the soldering process of the chip resistors on the copper metapatterns could be automated, fabrication errors might be caused during the process. To overcome this issue, inkjet printing methods have been proposed, which utilize silver nanoparticle inks [7,11,17]. Even though the fabrication processes are simple and based on conventional printing methods using cartridges filled with ink, the relatively slow speed of printing could cause an efficiency issue especially for a low sheet resistance [9]. Screen printing methods could be an alternative to the inkjet printing methods from the perspective of manufacturing efficiency. However, optimal sintering time and temperature have to be determined for the printed metapatterns [8,18,19]. Laser direct writing techniques could provide another solution; however, previous studies show that additional processes are needed to embed ferro ferric oxide into the graphene [9], or metasurface unit cells have to be connected with each other to transfer them on the dielectric substrate [20].

In this paper, we propose applying a metasticker to MAs that can be fabricated in a simple manner and reduces the reflectance by more than 10 dB in a broad BW for the normal incidence. To this end, a square-fractal ring is chosen as a metapattern to guarantee a large amount of design freedom, which could be designed optimally by using a genetic algorithm (GA). After designing an optimal one, an indium-tin-oxide (ITO) film is cut using a laser-cutting machine for the metapatterns, and flakes of them are recombined on a film with two adhesive sides. Then, the metasticker is attached on an acrylic substrate blocked by a copper sheet. From the measured results of the finalized total structure, which the fabrication tolerance by the laser-cutting machine compensates for, a broad −10 dB reflectance BW is confirmed from 4.39 to 7.51 GHz for the normal incidence, and its fractional BW is 52.44%. Moreover, a fractional BW of 4.35% is measured for a wide range of incident angles from 0° to 60° for both the TE and TM polarizations simultaneously.

## 2. Design Method

**Figure 1:**
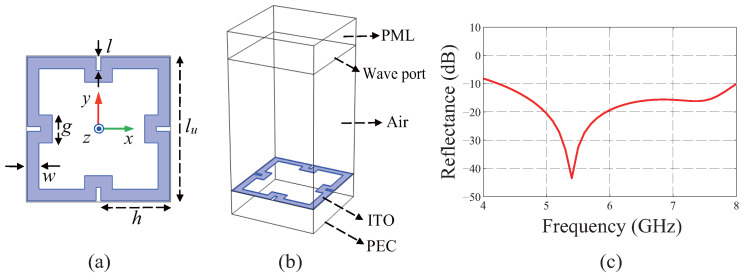
Schematics and a simulation result of the proposed metasurface absorber (MA). (**a**) Square-fractal ring pattern. (**b**) Simulation setting. (**c**) Simulated reflectance.

To design a broadband MA with a high degree of structural freedom, a square-fractal ring is chosen, as shown in Figure 1a. The structure has an advantage: it enables achieving dual-band response based on duplicated patterns in different scales [21]. In addition, based on a meandering-like current path, it has design freedoms, including controlling the self-inductance of the pattern by adjusting the length of it as well as adjusting the capacitance by changing the gaps between the corners of the pattern. As depicted in Figure 1a, the dimensions of the metapattern are determined by using four parameters: *w*, *l*, *h*, and *g*. To determine the optimal lengths of them, a GA is applied by using bit sequences which consist of 29 bits sectionalized by four groups. The first group composed of 5 bits is matched to the parameter *w*, and the other three groups equally composed of 8 bits are matched to *l*, *h*, and *g* sequentially. The geometric parameters could be determined with the millimeter unit by using decimal numbers multiplied by 0.1, which are converted from the binary ones included in the groups.

As the first step of the GA, 29-bit sequences are generated randomly by using Matlab R2022a. The number of sequences is determined by the number of the bits included in them, which could guarantee the diversity of the sequences sufficiently [8,14,16,19,21,22]. To prevent errors that break the square fractal metapattern, bit sequences that satisfy the following conditions are selected. To make a trough at each side of the square ring whose width is at least 0.4 mm, conditions of g≥2w+0.4 mm and l>w are considered where w≥0.2 mm. To prevent connection between the inner corners of the ring, a condition h>l+2w is applied. In order for a gap of at least 0.2 mm to remain between the patch and the outer boundary of the unit cell, a condition h+(g−2w)/2≤12.3 mm is applied when the size of the square unit cell $l_{u}$ is 25 mm. The size of 25 mm is equal to $\lambda_{0}/2$, where $\lambda_{0}$ indicates the wavelength in free space at 6 GHz. The condition prevents the generation of the backward grating lobe especially for oblique incidences at which scatterings from MA might be not eliminated sufficiently [23].

Then, full-wave simulations are conducted by using 29 pairs of the four geometric parameters decoded from the bit sequences. For the full-wave simulations, one of the commercialized finite-element-method solvers, Comsol Multiphysics 6.1, is used. Figure 1b shows the simulation setting for an MA that consists of an acrylic substrate whose top and bottom surfaces are covered by a metapattern composed of ITO and the perfect electric conductor (PEC), respectively. The thickness and the relative permittivity of the substrate are 7 mm and 2.6−i0.01, respectively. The thickness of the substrate is determined to satisfy $\lambda_{g}/4$, where $\lambda_{g}$ indicates the wavelength inside the medium at the center frequency of the target band, 6 GHz. The condition enables a constructive interference between the incident and the reflected electric (E) fields from a PEC at the height where the ITO metapattern is located, which could induce the maximum electric current on it.

The simulation domain out of MA is filled with the air which is truncated by the perfectly matched layer (PML). The periodic boundary condition (PBC) is set to the side boundaries of the entire setting. At the boundary between the air and PML, the wave port is allocated, which irradiates the plane EM wave. The polarization is set to the TE one of which the E field is along with the *y* axis shown in Figure 1a. To save computational resources as well as reduce the simulation time, the material properties of ITO are set on the two-dimensional (2D) metapattern by using the transition boundary condition (TBC) [24]. By referencing the previous studies utilizing carbon metapatterns whose sheet resistances is 100 Ω/sq [8,19], an ITO film is chosen whose sheet resistance $R_{s}$ ranges from 80 to 120 Ω/sq. By averaging the measured sheet resistances by the four-point-probe method, the sheet resistance $R_{s}$ is determined to be 93.54 Ω/sq. The thickness $t_{ITO}$ and the relative permittivity $\epsilon_{r}$ of the ITO are set to 58.6 nm [25] and $0.59\times 10^{6}$ [26], respectively. From $R_{s}$ and $t_{ITO}$, the conductivity $\sigma_{ITO}$ can be estimated as 182.43 kS/m based on the relation of $\sigma_{ITO}=1/\left(R_{s}\times t_{ITO}\right)$ [8,19].

Among the 29 pairs of the geometric parameters, the best two pairs are selected with the minimum figure of merit (FOM) values. An FOM is defined as

(1) $$\mathrm{FOM}=\frac{1}{3}\sum_{i=1}^{3}R_{TE,\theta =0{}^{\circ},f_{i}},$$

where $f_{1}$, $f_{2}$, and $f_{3}$ indicate 4, 6, and 8 GHz, respectively. The vertical incident angle θ defined from the *z* axis is set to 0° for the normal incidence. To design a broadband MA covering a single-frequency band, i.e., C band, it is supposed that three frequencies are sufficient to find a single-resonance response [8,27], which guarantees minimal computational time. In addition, the reflectance is calculated only for the TE polarization owing to the symmetries of the square-fractal ring for the *x* and *y* axes, as shown in Figure 1a. The results calculated using the TM polarizations are the same as those using the TE ones especially for the normal incidences [8,14,16].

To evolve the two selected pairs of geometric parameters, they are encoded into two-bit sequences by transforming decimal numbers into binary ones by multiplying each parameter by 10. Then, the cross and mutating operations are applied on them. The cross operation that exchanges the bits between two bit sequences is applied 7 times which is a quarter of the number of bits [8,14,16,19,21,22], and the mutating one that converts randomly selected bits from 1 to 0 or vice versa is applied 2 times, which is 6.9% of the number of bits. The ratio is determined heuristically to be included in the range from 2.38% [19] to 8.06% [14,16] by referencing previous studies. The cross and mutating operations using the selected two bit sequences are repeated until they produce 29-bit sequences as a new generation. Next, the bit sequences are decoded into metapatterns to apply the same competition process to select the best two pairs of geometric parameters. By repeating the whole GA process 8 times, the FOM values of the two bit sequences are converged to 0.087. The finalized geometric parameters of *w*, *l*, *h*, and *g* in Figure 1a are 2, 2.3, 11.9, and 4.8 mm, respectively. From the simulated reflectance using the finalized parameters in Figure 1c, a broad −10 dB reflectance BW is confirmed from 4.24 to 8 GHz for the normal incidence whose fractional BW is 61.44%.

## 3. Experimental Verifications

**Figure 2:**
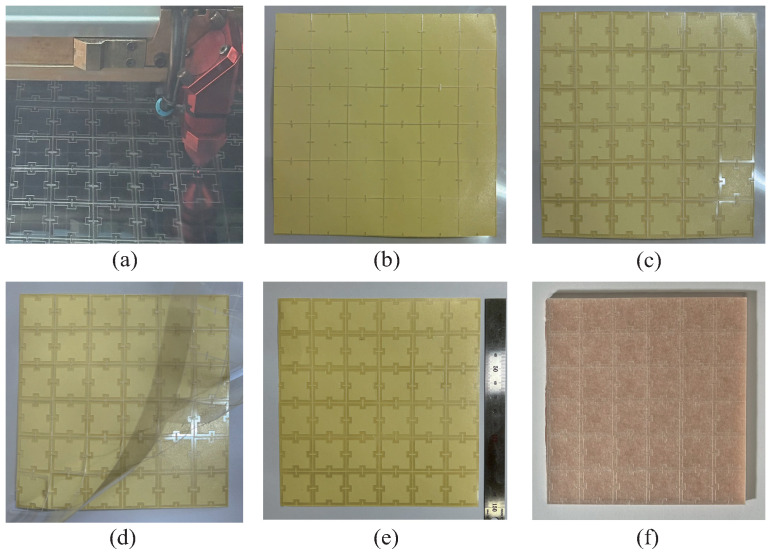
Fabrication process of metasurface absorber (MA). (**a**) Cutting stage of ITO film using a laser-cutting machine. (**b**) Prepared double-sided adhesive film with a frame and (**c**) assembled metapattern on it. (**d**) Process of removing the frame. (**e**) Fabricated metasticker. (**f**) Finalized MA.

To simplify the fabrication process of MAs, a metasticker is fabricated by using the finalized square-fractal metapattern. First, an ITO film whose average sheet resistance is 93.54 Ω/sq is cut by using a laser-cutting machine, as shown in Figure 2a. Second, the flakes of the metapattern achieved from the cutting process are assembled on a double-sided adhesive film by fitting them to a frame of Figure 2b, as shown in Figure 2c. The frame is the ITO film that remained after detaching the flakes from it. It enables aligning the metapatterns parallel to each other by maintaining the designed gap between the metapatterns. The entire sample consists of 6 by 6 unit cells, whose size is 150 × $150{\;\mathrm{mm}}^{2}$. Third, the frame is removed, as shown in Figure 2d, and a cover film is attached on the metapatterns to protect them from environmental damage as shown in Figure 2e. At last, the fabricated metasticker is attached on the dielectric substrate whose backside is blocked by a copper sheet using the opposite side of the adhesive film. Figure 2f shows the total structure of the fabricated MA.

**Figure 3:**
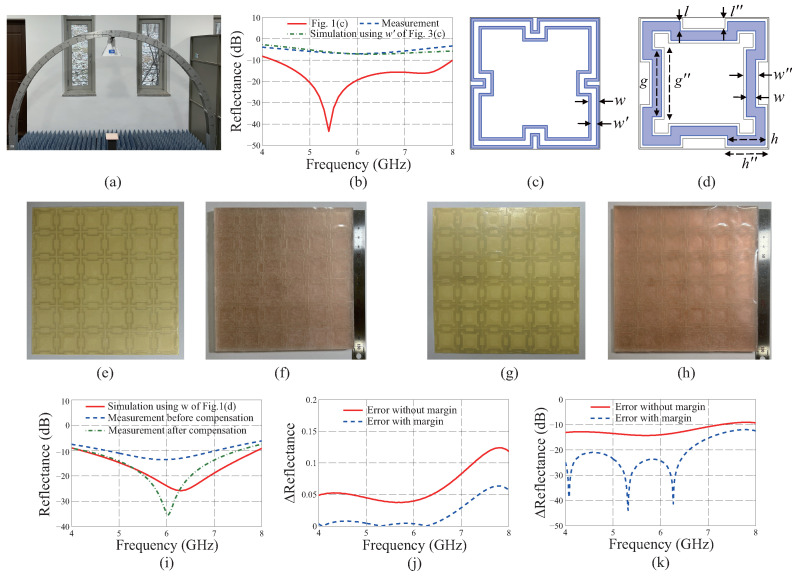
Metasurface absorber (MA) before and after compensating for the fabrication tolerance using the laser-cutting machine. (**a**) Measurement setting for reflectance of MA for the normal incidence. (**b**) Comparisons of simulated and measured results before compensation. (**c**) A schematic of Figure 1a with damaged regions by the laser-cutting machine. Blue color: damaged areas. (**d**) MA designed with margin. Blue color: redesigned metapattern. Black lines around the metapattern: marginal areas. (**e**) Fabricated metasticker and (**f**) MA without margin. (**g**) Fabricated metasticker and (**h**) MA with margin. (**i**) Comparison of simulated result of redesigned MA and measured results of it before and after compensation. Errors between the measured results and the simulated one in (**j**) linear and (**k**) logarithmic scales.

The reflectance of the fabricated MA is measured using an antenna system composed of two broadband double-ridged horn antennas connected to the Keysight (Santa Rosa, CA, USA) E5080B vector network analyzer (VNA). Before measurement, a cable calibration for the VNA is conducted. For the normal incidence, the reflectance is calculated by dividing ${\left|S11\right|}^{2}$ (measured from the MA) by that from the copper plate whose size is the same as that of the MA, 150 × $150{\;\mathrm{mm}}^{2}$, where ${\left|S11\right|}^{2}$ denotes the ratio between the incident power and the reflected one. Figure 3a,b show the measurement setting and the measured reflectance for the normal incidence, respectively. From Figure 3b, a quite large discordance is found between the measured and the simulated results, which is due to the ITO film being damaged during the laser-cutting process. To investigate its fabrication tolerance, a simulation study is conducted by changing the width of the metapattern to fit the simulated result to the measured one. As a consequence, the simulated result is well matched to the measured one when the width is reduced from w=2 mm to $w^{\prime }=1$ mm, as shown in Figure 3b. The reductions of the width from the outer and the inner boundaries of the metapattern are found to be 0.5 mm, which are depicted as two blue lines in Figure 3c.

To compensate for the degraded performance, the metapattern is redesigned by considering the reduction of the width *w*. To this end, the ITO film is changed to another one whose sheet resistance is lower than that of the original one to minimize the increase in the resistance of the damaged metapattern. The averaged sheet resistance of the alternative film is measured as 39.12 Ω/sq. As the sheet resistance changes, the thickness of the ITO film is substituted to be 114.1 nm [25]. Accordingly, the conductivity is calculated as 224.03 kS/m. In addition, the conditions g≥2w+0.2 mm, h>l+2w, and h+(g−2w)/2≤12.3 mm for the selection of the bit sequences are modified to g≥2w+1.4 mm, h>l+2w+1 mm, and h+(g−2w)/2≤11.8 mm, respectively, to guarantee. Then, the best two-bit sequences are found from randomly generated ones, and the GA is applied 6 times repeatedly until the FOM of the bit sequences are converged to 0.083 using the process described in Section 2.

As a result, the modified geometric parameters of *w*, *l*, *h*, and *g* of Figure 3d are found to be 1.8, 1.8, 7.2, and 12.7 mm, respectively. A blue line in Figure 3d shows the redesigned metapattern while the black lines surrounding the blue color indicate the modified version including the margin. The compensated dimensions of $w^{\prime \prime }$, $l^{\prime \prime }$, $h^{\prime \prime }$, and $g^{\prime \prime }$ of the finalized metapattern shown in Figure 3d are 2.8, 1.8, 8.2, and 13.7 mm, respectively. Figure 3e,f show the metasticker and MA without the margin, respectively, that are fabricated by following the same process described using Figure 2. Figure 3g,h show the metasticker and MA with the margin, respectively. Figure 3i shows a comparison between the simulated and measured results for the normal incidence of the TE polarization, which clearly confirms that the deteriorated measured result is sufficiently compensated for by applying the margin. To verify the effectiveness of the proposed method quantitatively, the magnitudes of the differences between the simulated reflectance and the measured ones are calculated in the linear scale as shown in Figure 3j. Figure 3k shows the differences in the logarithmic scale. From the results, the effect of the margin of Figure 3d is clearly confirmed.

**Figure 4:**
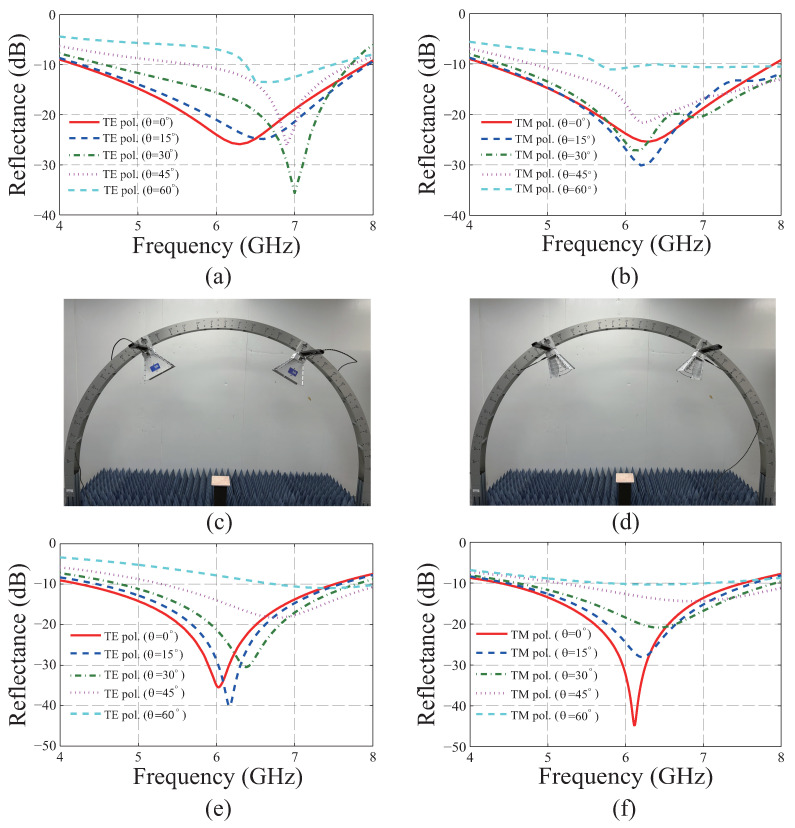
Simulated and measured reflectance of the redesigned metasurface absorber. Simulated reflectance for (**a**) transverse electric (TE) and (**b**) transverse magnetic (TM) polarizations. Measurement settings of oblique incidences with θ=30° for (**c**) TE and (**d**) TM polarizations. Measured reflectance with various incident angles for (**e**) TE and (**f**) TM polarizations.

Figure 4a,b show the simulated reflectance for TE and TM polarizations with the incident angles from 0° to 60° with an interval of 15°. For the normal incidence, a −10 dB reflectance BW is simultaneously confirmed from 4.21 to 7.9 GHz for the TE and TM polarizations, whose fractional BW is 60.94%. For the incident angles from 0° to 60°, −10 dB reflectance BWs are simultaneously confirmed from 6.37 to 7.49 GHz and from 5.66 to 7.9 GHz whose fractional BWs are 16.16% and 33.04% for the TE and TM polarizations, respectively. For both of the polarizations with the full-incident angles, the reflectance simultaneously remains below −10 dB from 6.37 to 7.49 GHz, and its fractional BW is 16.16%. To verify the absorbing performance experimentally, the same measurement setting of Figure 3a is utilized for the normal incidence.

To measure the specular reflectance for the oblique incidence scenarios, the measurement settings shown in Figure 4c,d are used for the TE and TM polarizations, respectively. The specular reflectance is evaluated by dividing ${\left|S21\right|}^{2}$ of the MA with that of the copper plate where ${\left|S21\right|}^{2}$ indicates the ratio between the incident power from port 1 and the specularly reflected power received by port 2. To improve the accuracy of the measurement, the time-gating function of VNA is utilized to capture the main lobe reflected from the sample. To this end, the location of the rectangular window on the time domain is predetermined to include the reflected main lobe from the copper plate, which is the reference target for the measurement. Figure 4e,f show the measured reflectance for TE and TM polarizations with the incident angles from 0° to 60° increased by an interval of 15°. For the normal incidence, −10 dB reflectance BW is simultaneously confirmed from 4.39 to 7.51 GHz is for the TE and TM polarizations, whose fractional BW is 52.44%. For the angles from 0° to 60°, a −10 dB reflectance BW is simultaneously confirmed from 6.74 to 7.51 GHz and from 5.77 to 7.04 GHz, and their fractional BWs are 10.81% and 19.83% for the TE and TM polarizations, respectively. For both of the polarizations with the full-incident angles, the reflectance simultaneously remains below −10 dB from 6.74 to 7.04 GHz, and the fractional BW is 4.35%.

**Figure 5:**
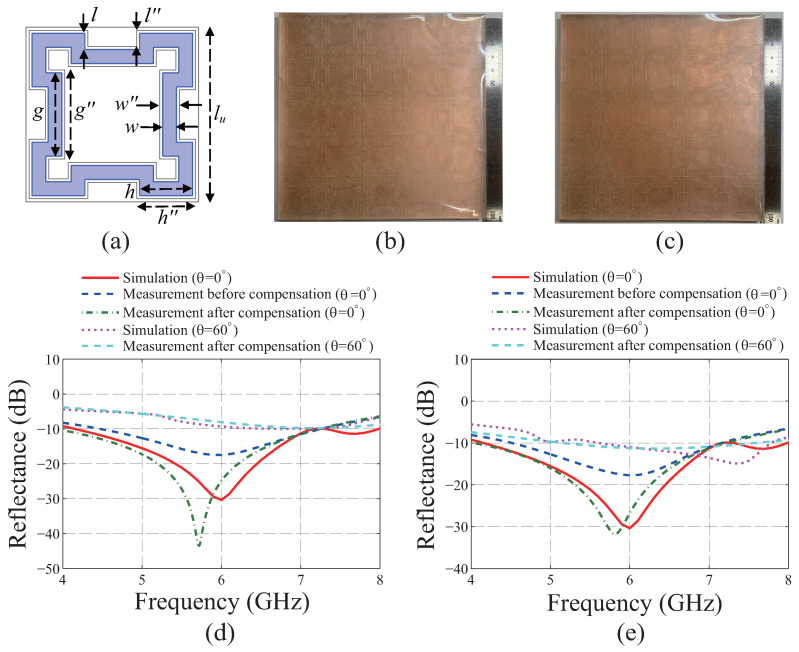
Verification of reproducibility of the proposed design method. (**a**) Unit cell of metasurface absorber (MA) whose size is 30 mm. Fabricated MA (**b**) without margin and (**c**) with margin. Simulated and measured results for the normal and the incident angle θ of 60° for the (**d**) transverse electric and (**e**) transverse magnetic polarizations.

To verify the reproducibility of the proposed design method, another MA is designed as shown in Figure 5a whose unit cell size, $l_{u}$, is 30 mm. The size of the unit cell is increased from 25 mm because it guarantees more diverse combinations of the geometric parameters while considering the margin. For the design, the condition h+(g−2w)/2≤11.8 mm for the original MA whose $l_{u}$ is 25 mm is modified to h+(g−2w)/2≤14.3 mm while the other conditions are maintained. By applying the whole GA process 7 times under the modified condition, the FOM values of the two bit sequences are converged to 0.074. From the finalized result, the geometric parameters of *w*, *l*, *h*, and *g* are found to be 2.4, 2.8, 9.2, and 14.3 mm, respectively. By considering the marginal width of 0.5 mm from the inner and the outer boundaries of the metapattern, the compensated dimensions of $w^{\prime \prime }$, $l^{\prime \prime }$, $h^{\prime \prime }$, and $g^{\prime \prime }$ in Figure 5a are determined to be 3.4, 2.8, 10.2, and 15.3 mm, respectively. Figure 5b,c show the fabricated MA without and with the margin, respectively. Even though it is found that the reflectance of the redesigned MA is not reduced below −10 dB for the TE polarization with the incident angle θ of 60°, as shown in Figure 5d, the effectiveness of the proposed method could be verified by the results for the normal incidences in Figure 5d,e whose polarizations are TE and TM, respectively. From the results, it is confirmed that the gaps between the simulations and the measurements are reduced effectively by the margin.

Table 1 shows the comparisons between the simulated and the measured −10 dB reflectance BW of the proposed MA and the previously reported ones utilizing single-layer metapatterns. From comparing the fractional BW for the normal incidence, it is confirmed that the MA based on graphene-assembled films [28] is the widest among the MAs, as summarized in Table 1. However, a drawback is found: the range of the incident angle is limited to 30°. On the other hand, a wide-angle MA based on a crescent shape resonator [13] has been reported whose overlapped BW is maintained with the incident angle ranging from 0° to 60° for both TE and TM polarizations. However, it also has a limitation in that the BW is very narrow, and the overlapped BW is not confirmed from the measurements. Although the measured overlapped BW is reported using an eight-circular sector [12], the BW is still very narrow. Based on the proposed MA, a 4.35% fractional BW is achieved in a wide incident angle range of up to 60°. Even though the BW for the oblique incidence scenario is narrower than that of an atypical MA utilizing chip resistors [14], the BW of the proposed MA is much wider than that from the previous study for the normal incidence. In addition, it is found that the relative thickness of the proposed MA for the wavelength inside the substrate at the lowest frequency of the measured BW $\lambda_{l}$ is thinner than that of the previous study.

## 4. Conclusions

The design and fabrication strategies are proposed for the broadband MA based on the concept of a metasticker, which can be attached on a dielectric substrate blocked by a copper sheet. To extend the absorption BW as broadly as possible, the square-fractal-ring metapattern is chosen and optimally designed by using a GA. The metasticker has a great advantage in that it can be fabricated easily by cutting the ITO film using a laser-cutting machine and assembling the metapattern on the double-sided adhesive film. To compensate for the fabrication tolerance of the cutting process, the margin of 0.5 mm found by the simulation studies is added on both the left and right sides of the optimized design. From the measured results of the finalized total structure of the MA, a broad −10 dB reflectance BW is confirmed from 4.39 to 7.51 GHz for the normal incidence whose fractional BW is 52.44%. Moreover, a fractional BW of 4.35% is measured in a wide incident angle range from 0° to 60° for both the TE and the TM polarizations simultaneously. There is still room for improvement in the bandwidths in terms of increasing the number of frequencies included in the FOM for the normal incidence and applying a weighting factor to the reflectance of the TE polarization with the angle θ of 60° for the oblique incidence [14]. In addition, from the perspective of the manufacturing process, a cutting method utilizing diamond tools [29] could be an alternative to improve the structural accuracy of the metapattern. The proposed method may provide a powerful solution to design and fabricate broadband MAs which could be easily attached on antenna systems [30] that need to prevent interference from EM waves.

## Figures and Tables

**Table 1:** Comparisons of simulated and measured −10 dB reflectance bandwidths (BWs) of single-layer metasurface absorbers (MAs) for normal and various incident angle ranges. The BWs are defined as the intersected ranges of the BWs for both transverse electric and transverse magnetic polarizations. The incident angle, the wavelength of the substrate at the lowest frequency of the measured BW for the normal incidence, and the thickness of the MA are indicated by θ, $\lambda_{l}$, and $d_{t}$, respectively. NA: not applicable.

	Incident Angle θ	−10 dB Reflectance BW				$d_{t}$	$d_{t}/\lambda_{l}$
		Simulation		Measurement			
	**deg**	**GHz**	**%**	**GHz**	**%**	**mm**	
Figures 4 and 7 of [12]	0	9.2–9.33	1.4	9.19–9.31	1.3	0.8	0.05
	0–60	9.25–9.28	0.32	9.21–9.31	1.08		
Figures 5 and 9 of [13]	0	5.13–5.22	1.74	5.18–5.31	2.48	1.6	0.057
	0–60	5.14–5.18	0.78	NA			
Figures 3 and 4 of [14]	0	9.22–11.05	18.06	9.5–11.56	19.56	3.6	0.24
	0–60	10.80–11.21	3.73	9.74–11.26	14.48		
Figures 2 and 3 of [28]	0	4.24–17.9	123.4	4.03–18.17	127.39	5.8	0.146
	0–30	11.11–18.23	48.53	NA			
This work	0	4.21–7.9	60.94	4.39–7.51	52.44	7	0.165
	0–60	6.37–7.49	16.16	6.74–7.04	4.35		

## Data Availability

The original contributions presented in this study are included in the article. Further inquiries can be directed to the corresponding author.
